# Benefits of Immobilized Bacteria in Bioremediation of Sites Contaminated with Toxic Organic Compounds

**DOI:** 10.3390/microorganisms13010155

**Published:** 2025-01-14

**Authors:** Emanuel Gheorghita Armanu, Simone Bertoldi, Łukasz Chrzanowski, Irina Volf, Hermann J. Heipieper, Christian Eberlein

**Affiliations:** 1 Department of Molecular Environmental Biotechnology, Helmholtz Centre for Environmental Research—UFZ, 04318 Leipzig, Germany; gheorghita-emanuel.armanu@student.tuiasi.ro (E.G.A.); simone.bertoldi@ufz.de (S.B.); christian.eberlein@ufz.de (C.E.); 2 Department of Environmental Engineering and Management, “Gheorghe Asachi” Technical University of Iasi, 73A Prof. D. Mangeron Blvd., 700050 Iasi, Romania; 3 Institute of Chemical Technology and Engineering, Poznan University of Technology, 60-965 Poznan, Poland; lukasz.chrzanowski@put.poznan.pl

**Keywords:** BTEX, contaminants of emerging concern, polycyclic aromatic hydrocarbons, nano-pollutants, microbial immobilization, support materials, microcolonies, solvent tolerance, biodegradation

## Abstract

Although bioremediation is considered the most environmentally friendly and sustainable technique for remediating contaminated soil and water, it is most effective when combined with physicochemical methods, which allow for the preliminary removal of large quantities of pollutants. This allows microorganisms to efficiently eliminate the remaining contaminants. In addition to requiring the necessary genes and degradation pathways for specific substrates, as well as tolerance to adverse environmental conditions, microorganisms may perform below expectations. One typical reason for this is the high toxicity of xenobiotics present in large concentrations, stemming from the vulnerability of bacteria introduced to a contaminated site. This is especially true for planktonic bacteria, whereas bacteria within biofilms or microcolonies have significant advantages over their planktonic counterparts. A physical matrix is essential for the formation, maintenance, and survival of bacterial biofilms. By providing such a matrix for bacterial immobilization, the formation of biofilms can be facilitated and accelerated. Therefore, bioremediation combined with bacterial immobilization offers a comprehensive solution for environmental cleanup by harnessing the specialized metabolic activities of microorganisms while ensuring their retention and efficacy at target sites. In many cases, such bioremediation can also eliminate the need for physicochemical methods that are otherwise required to initially reduce contaminant concentrations. Then, it will be possible to use microorganisms for the remediation of higher concentrations of xenobiotics, significantly reducing costs while maintaining a rapid rate of remediation processes. This review explores the benefits of bacterial immobilization, highlighting materials and processes for developing an optimal immobilization matrix. It focuses on the following four key areas: (i) the types of organic pollutants impacting environmental and human health, (ii) the bacterial strains used in bioremediation processes, (iii) the types and benefits of immobilization, and (iv) the immobilization of bacterial cells on various carriers for targeted pollutant degradation.

## 1. Introduction

Environmental pollution by organic compounds poses significant challenges to ecosystems and human health. These pollutants often originate from their widespread use in industrial processes, manufacturing, and everyday activities. Organic compounds can contaminate soil, groundwater, and air through improper disposal, leaks, or accidental spills [1,2]. Common pollutants include hydrocarbons, agricultural chemicals such as pesticides and herbicides, synthetic dyes, and surfactants, as well as increasingly significant contaminants like polymer-related compounds, nano-plastics, plasticizers, stabilizers, nanoparticles, and perfluorinated organic compounds such as per- and polyfluoroalkyl substances (PFASs). Unfortunately, the list of contaminants is continuously expanding, with the chemical industry being the major contributor [3,4]. Currently, it is reported that more than 160 million compounds have been produced, most of which were created in recent decades [5]. Some recent inventories estimate that the real number of chemicals surpasses 350 million. The United States alone introduces approximately 1500 new substances each year. Consequently, the number of pollutants will continue to grow alarmingly, leading to increasing risks to global ecosystems and human health [6].

**Figure 1:**
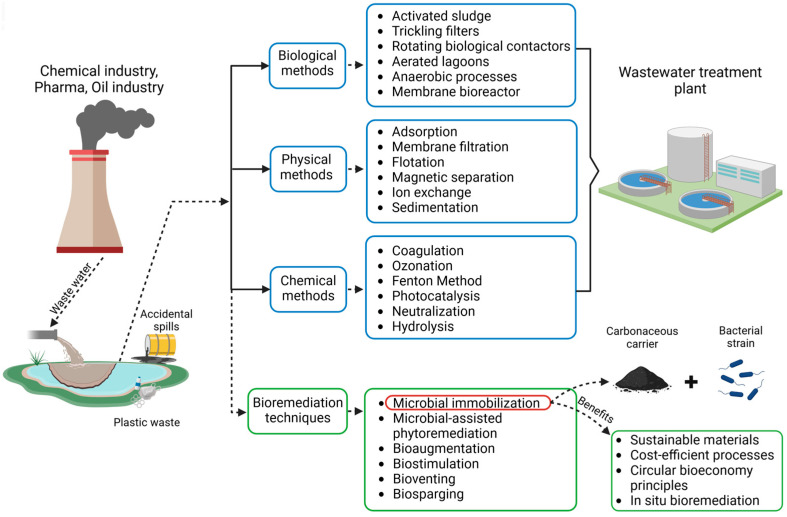
An overview of the most important methods used for organic compound/pollutant removal from contaminated sites.

Once released into the environment, these chemicals can persist for extended periods, posing risks to both terrestrial and aquatic ecosystems [7]. Efforts to mitigate environmental contamination such as bioremediation and physicochemical treatments are employed to reduce the impacts of contamination and restore affected ecosystems [8]. In common perception, the use of biological methods to remove such pollutants is considered an environmentally friendly alternative to physicochemical methods. In practice, however, a combination of both techniques (Figure 1) is often applied [9]. Physicochemical methods are highly effective for rapidly removing large amounts of pollutants, but as pollutant concentrations decrease, these methods become increasingly expensive and less effective [10]. In such cases, bioremediation offers an efficient solution for the complete elimination of residual contaminants.

Bioaugmentation is a common bioremediation technique and it involves accelerating the degradation of contaminants by introducing specific microorganisms as mixed cultures, communities, or specialized consortia. However, the success of this approach is often limited by various in situ challenges that increase microbial vulnerability [11]. Among these challenges, the toxicity of pollutants is a major factor contributing to the inability of introduced bacteria to survive or thrive under the specific environmental conditions of the site [12]. Moreover, competition with indigenous microorganisms or exposure to other toxic substances present alongside the main pollutant at the site can further inhibit bacterial activity [13]. In addition, fluctuations in (harsh) environmental conditions such as temperature, pH, nutrients, and electron acceptor availability can also impact the effectiveness of bacterial bioremediation efforts. Planktonic microorganisms, which are typically introduced during remediation, often struggle to survive under such harsh conditions. Enhancing their survival and resistance through immobilization techniques can significantly improve their efficacy [14]. It is known that bacteria that grow in microcolonies and biofilms have considerable advantages over the planktonic way of life. The formation of cell aggregates is an important factor for increased tolerance and robustness. The biofilm matrix comprises cells, extracellular polymeric substances (EPSs), proteins, DNA, and other macromolecules [15]. The formation of (outer) membrane vesicles facilitates biofilm formation due to the increased hydrophobicity of the cell surface [16,17,18]. Cells within the microenvironment formed by biofilms are shielded, enhancing their ability to endure and withstand challenging environmental conditions such as exposure to antimicrobial agents, heat, or solvents [18,19,20]. This was also reported for aromatic compounds such as phenol and 4-chlorophenol [21,22]. Hence, in the process of bioaugmentation/bioremediation, adding bacterial microcolonies/biofilms is more promising due to their robustness [23,24].

One innovative application of bacterial biofilms is their use in bioaugmentation strategies, where biofilms are immobilized on carriers to enhance their stability and effectiveness in pollutant degradation. Immobilization significantly enhances these introduced bacteria’s survival and adaptation by providing an extended period for acclimatization within the native microbial ecosystem [25,26]. Very recently, it was shown that the bioaugmentation of an experimental site polluted with ibuprofen failed when planktonic cultures of a previously isolated bacterium were added, but was successful after adding gravel containing immobilized cells of the bacteria [27,28]. This approach can often eliminate the need for remediation based on physicochemical methods, significantly reducing environmental cleanup costs. However, the immobilization matrix required for this type of bacterial lifestyle should be well selected in order to make optimum use of the microbial potential, achieve a high level of pollutant degradation, and be economically feasible. Among physical properties like structural stability and providing a protective habitat, adhesion and nutrient capture are crucial parameters [26,29].

This review provides a summary on the state-of-the art (i) types of organic pollutants impacting environmental and human health, (ii) bacterial strains used in bioremediation processes, (iii) carriers produced via thermochemical biomass conversion, (iv) types and benefits of immobilization, and (v) immobilization of bacterial cells on various carriers for targeted pollutant degradation.

## 2. Types of Pollutants That Can Affect the Environment and Human Health

To date, from the 160 million chemicals that have been produced worldwide, around 60,000 are actively used in global trade. Among these, 6000 chemicals account for more than 99% of the global total volume that is used commercially [5]. The constant release of new compounds into the environment has led to various short- and long-term effects [30]. Industries such as plastics, fuel and energy, fashion, dyes, antibiotics, pesticides, construction, transport, mining, and chemical manufacturing, as well as intensive agriculture, significantly impact air, water, and soil, leading to irreversible damage to ecosystems and human well-being [31,32,33,34].

Pollutants, both inorganic and organic, are now present in every environment, posing risks of toxicity and bioaccumulation [35]. Among these, organic pollutants (Table 1) represent the most hazardous category due to their diversity, persistence in the environment, and ability to bioaccumulate along the trophic chain, which has made them a major concern in recent decades [36,37]. The molecular structure, biological activity, and use/function of organic pollutants directly influences their reactivity, toxicity, and environmental fate. Therefore, a classification of the key groups include anthropogenic organic pollutants; substituted monoaromatic and polycyclic aromatic hydrocarbons (PAHs) [38]; polychlorinated biphenyls (PCBs) [39]; dioxins and furans [40]; polyfluoroalkyl substances (PFASs) [41]; volatile organic compounds (VOCs) [42]; pesticides [43]; pharmaceuticals [44]; personal care products [45]; and endocrine disrupting chemicals (EDCs) [46], among others. Organic pollutants can exhibit high persistence in the environment and organisms due to their chemical stability especially when compound structures are stabilized by resonance effects or by the combination of carbon atoms with more electronegative elements such as F, Cl, or Br; these interactions confer high resistance to degradation like in the case of PFASs [47,48]. The structural complexity of many organic pollutants, such as phthalate alkyl esters and chlorinated compounds (e.g., PCBs), make them resistant to chemical, biological, or photolytic degradation [49]. Environmental factors such as temperature, pH, and water availability play a significant role in determining the stability of these chemicals and influence their biodegradation rates [2]. For example, cold, anaerobic, or nutrient-poor conditions often lead to slower biodegradation due to reduced microbial growth and metabolism rates. In such conditions, the limited availability of oxygen, which provides the highest energy yield as an electron acceptor, significantly hampers degradation processes [50]. Among organic compounds, those with low volatility and high hydrophobicity cause the biggest threat to the environment due to their toxicity for living organisms. They accumulate in and disrupt cell membranes and essential metabolic processes such as respiration and cell growth, which can be inhibited. The toxicity of such compounds often correlates with the logarithm of its partition coefficient between octanol and water [51]. Despite their persistence, many organic pollutants, including hydrocarbons, can be degraded or transformed into less harmful compounds by microorganisms under suitable conditions. Hydrocarbons, particularly straight-chain hydrocarbons, remain an energy source for certain microorganisms due to their accessibility and relatively low energy input for degradation [52]. However, the hydrophobic nature of these compounds often causes them to adsorb onto soil or mineral pores, preventing hydrophilic water from washing them away and rendering them unavailable for degradation [53].

Nonetheless, the use of adapted microorganisms, particularly indigenous strains, offers a sustainable approach for biodegrading these pollutants. Such microbes can completely degrade or at least transform pollutants into less-toxic substances, providing a promising solution for addressing environmental contamination [47,52,54].

**Table 1:** Classification of pollutants, origin of their source, and their effect upon the environment and human body.

Class	Examples	Sources	Effects	References
Industrial solvents	Acetone, tetrachloroethylene, toluene, trichloroethylene, dichloro-methane, tetrachloroethylene	Dry cleaning, metal degreasing, paint thinners, adhesives, dye manufacturing, paints and glues	Damage human liver, kidney, neural and immune systems; increase the level of volatile organic compounds indoors or outdoors	[55,56,57]
Polycyclic aromatic hydrocarbons (PAHs)	Benzo [a] pyrene, naphthalene, anthracene, chrysene, biphenyl, fluorene, tetracene	Incomplete combustion, power generation, agricultural waste, rubber manufacturing	Risk of lung cancer; increases cardiovascular disease, hypertension, and myocardial infarction; soil and water contamination	[38,58,59,60]
Polychlorinated biphenyls (PCBs)	Aroclor 1254, Aroclor 1260, Ascarel, Phenoclor, Clophen	Leaks or releases from electrical transformers, fuel combustion, chemical wastewater disposal sites, agriculture	Effect the immune system and reproductive system, neurobehavioral deficits, dementia, reduce aquatic life	[39,61,62]
Dioxins and furans	2,3,7,8-tetrachlorodibenzo-*p*-dioxin, dibenzofurans, polychlorinated dibenzofurans	Incineration (waste), combustion, industrial materials and processes, volcanic eruptions	Influence marine and terrestrial organisms, effect tissues and cells, cancerogenic, effect reproductive system	[40,63,64]
Polyfluoroalkyl substances (PFASs)	Perfluorooctanoic acid, Perfluorooctanesulfonic acid	Oil exploitation activities, food packaging, chemical industry, cosmetics, ski wax, apparel	Alter the immune response, thyroid, breast cancer, liver damage, obesity	[65,66]
Volatile organic compounds (VOCs)	Benzene, toluene, xylene, formaldehyde	Paints, pesticide aerosol sprays, disinfectants, copiers, printers, markers, tobacco	Increase the risk of breast cancer, respiratory illnesses, leukemia, neural tube defects	[41,67,68]
Pesticides and herbicides	Dichlorodiphenyltrichloroethane, Chlordane, Aldrin, Glyphosate, Atrazine	Agriculture, industrial wastewater	Bioaccumulation in mammals; they can effect plant transpiration rate and plant growth	[69,70,71]
Pharmaceuticals and personal care products	Antibiotics, antidepressants, hormonal drugs, sunscreen agents	Pharmaceutical waste (e.g., expired and unused pills, body care and cleansing, cleansing pads, etc.), veterinary medicines, wastewater treatment plants	Increase antimicrobial resistance, contaminates soils and water bodies (eutrophication)	[72,73]
Endocrine-disrupting chemicals (EDCs)	Bisphenol A, phthalates, phenol, xenobiotics	Pharmaceuticals, estrogens and androgens, industrial chemicals, long-chain polymers, pesticides, plasticizers, organometals	Ovarian disorder, endocrine disruptor, interference with testosterone, sperm motility, testicular cancer, effect aquatic life	[74,75,76]
Fertilizers	Sewage sludge, green waste compost and mixed digestate	Agriculture, wastewater treatment plants, industrial plants	Alter the rhizosphere micro-ecological environment, cell inhibition, organ tumors, infections	[77,78,79]

## 3. Bacterial Strains Used in Bioremediation Processes

In environmental systems, microbial communities typically comprise diverse bacterial strains rather than isolated species. This biodiversity contributes to dynamic systems that respond adaptively to contamination [80,81].

Microorganisms, including bacteria, fungi, protozoa, and microalgae, possess effective mechanisms for degrading or transforming various compounds that serve as energy sources for metabolic processes. Among these, bacteria are particularly noteworthy due to their ubiquity, versatility, resilience, and adaptability across diverse environments [82,83].

Metabolically versatile microorganisms with significant environmental and biotechnological importance are Gram-positive bacterial genera, such as *Bacillus* (Firmicutes), *Mycobacterium*, *Arthrobacter*, and *Rhodococcus* (Actinobacteria), as well as Gram-negative ones like *Flavobacterium* (Bacteroidota), *Acinetobacter*, and *Pseudomonas* (Proteobacteria). They play a critical role in bioremediation processes due to their resistance and ability to degrade a wide variety of pollutants (e.g., PAHs, PCBs, EDCs, etc.) [84,85,86,87,88]. Below, some of the key bacterial genera commonly used in bioremediation processes and the specific pollutants they target are mentioned.

*Bacillus* are Gram-positive, rod-shaped, aerobic/anerobic bacteria and represent an important candidate for the degradation of PAHs and pesticides. The *Bacillus* genus is found in soil and water and has a diverse assemblage of predominant aerobic strains. Furthermore, it possesses a high resistance to temperatures and an increased metabolic adaptability in nutrient-depleted soils or wastewater [89,90]. *Bacillus amyloliquefaciens*, a plant growth promoter, could degrade benzene, toluene, ethylbenzene, and xylene (BTEX) compounds when applied individually in vitro [91]. Moreover, *Bacillus* was identified as one of the major oil-degrading bacterial genera in the context of the Deepwater Horizon oil spill [92]. Some *Bacillus* strains in situ can secrete biosurfactants (e.g., surfactin), which can enhance the solubility and bioavailability of hydrophobic pollutants, thus making them more accessible for degradation processes [93]. Techniques like bioaugmentation have shown promising results in accelerating pollutant breakdown and can increase crop tolerance to multiple abiotic stresses [94].

*Mycobacterium* strains are non-spore-forming, rod-shaped, aerobic, and opportunistic pathogens. Some strains exhibit a high biodegradation of PAHs, which are a major class of environmental pollutants [95]. Their slow growth is compensated by their ability to metabolize recalcitrant compounds (e.g., phenanthrene and naphthalene) under aerobic conditions [96]. Also, they can produce specialized enzymes such as mono-oxygenase and dioxygenase to degrade aromatic compounds, which break into simpler molecules, aiding bioremediation efforts. Another important role in their robustness against PAHs is represented by the hydrophobic cell walls and versatile catabolic pathways [94,97].

*Arthrobacter* are obligate aerobes bacteria that can utilize various organic compounds, particularly persistent contaminants (pesticides, PAHs, and PCBs), which act as a source of energy for their metabolic processes. *Arthrobacter* exhibits a high tolerance to various environmental stresses, such as extreme temperatures, osmotic pressure shifts, long-term starvation, oxidative stress, pH fluctuations, and an excess of metal ions [98,99]. Arthrobacter is really efficient at degrading triazines, organophosphorus, alkaloids, benzene, etc., due to the presence of various degrading genes located on the plasmids. An example of such a gene is the atrazine-degrading gene, such as that found in the *Arthrobacter* sp. AK-YN10 plasmid [100,101].

*Rhodococcus* are sphere-shaped, obligate aerobic and non-motile bacteria that have high efficiency in the bioremediation and bioconversion of recalcitrant organic pollutants (organochlorine compounds, hydrocarbons, dyes, antibiotics, and pesticides) and pharmaceutical contaminants [102,103]. Their metabolic versatility and enzymatic potential are attributed to an array of mono-oxygenase and dioxygenase enzymes, which enable them to break down complex compounds in various environmental conditions [104]. These bacteria are particularly noted for their potential in bioaugmentation and immobilized cell technologies, which enhance pollutant (hydrocarbons, PAHs, and PCBs) breakdown in soil and water [105].

*Flavobacterium* is a genus of rod-shaped bacteria, which are motile by gliding, strictly aerobic, and omnipresent within soil and water bodies [106]. They are also known to be resistant in extreme conditions (e.g., in the Arctic region) and tolerant to nutrient-depleted environments [107]. *Flavobacterium* can degrade various organic pollutants, including pesticides, hydrocarbons, and azo dyes [108]. For example, *Flavobacterium johnsoniae* has been identified to degrade naphthalene, while other *Flavobacterium* strains effectively break down phenols and polyphenols [109,110].

*Acinetobacter* strains are predominantly found in soil and water bodies. The genus is known for its effectiveness in degrading hydrocarbons, being a valuable candidate in oil spill bioremediation [111]. Furthermore, this bacterium appears to play a key role in hydrocarbon-degrading communities, even in environments contaminated with both aromatic hydrocarbons and metals like copper, zinc, and lead [112]. One significant advantage of *Acinetobacter* in bioremediation stays in its ability to degrade pollutants through various enzymatic pathways, often in combination with other microorganisms; this makes them valuable in microbial consortia systems [113,114].

*Pseudomonas* strains, strictly aerobic bacteria, are widely distributed in many environmental niches [115]. *Pseudomonas* strains are key players in the biodegradation of a wide range of organic pollutants, such as phenol, trichloroethane, benzene, and toluene [116]. Techniques like biostimulation and bioaugmentation are commonly used to enhance *Pseudomonas* activities in contaminated sites, improving the efficiency of pollutant degradation. Integrated bioremediation approaches are becoming increasingly important to manage large-scale hydrocarbon contamination sustainably [87]. *Pseudomonas* strain ADP, an isolate coming from a herbicide spill site, is capable of metabolizing high concentrations of atrazine [117].

It has to be mentioned that discrepancies between in vitro studies and in situ bacterial tests can arise because lab conditions are controlled and simplified, while natural environments are complex and variable. Factors like nutrient availability, interactions with other organisms, and fluctuating physical conditions in situ significantly influence bacterial behavior, which is often missed in lab studies. These differences highlight the need to validate lab findings in real-world contexts to ensure accurate applications in environmental settings.

## 4. Principles and Benefits of Immobilization

**Figure 2:**
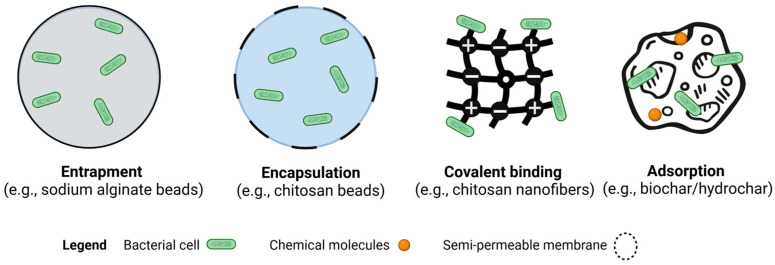
An overview of the main bacterial immobilization methods on various support materials.

Microbial cell immobilization involves fixing microbial cells onto carriers or support materials through physical or chemical methods. Physical methods, such as entrapment and encapsulation, bind cells irreversibly, creating stable immobilized systems [118,119]. Immobilized bacteria offer numerous advantages, including enhanced stability, reusability, efficiency, targeted application, and environmental safety [120]. The immobilization process forms a protective barrier around microbial cells, enhancing their resilience to adverse environmental conditions and prolonging their functional lifespan. Additionally, immobilized bacteria can be easily recovered and reused multiple times, reducing costs and increasing efficiency in microbial processes [33,121]. Below, the main principles of microbial cell immobilization are outlined. Additionally, Figure 2 and Table 2 give an overview of the principles and benefits of immobilization.

### 4.1. Physical Immobilization Methods

Entrapment captures cells within a support matrix (e.g., sodium alginate beads) or hollow fiber, creating a barrier to protect cells from external conditions and reduce leakage (Figure 2). Common carriers for entrapment include chitosan, alginate, and various synthetic polymers [120,129]. Encapsulation, a form of entrapment, envelops biological components within semi-permeable membranes, allowing pollutants to diffuse into the matrix (chitosan beads) while providing a barrier [123].

### 4.2. Chemical Immobilization Methods

Covalent binding involves forming covalent bonds with chemical additives, which is widely used for enzyme immobilization [125]. For example, porcine pancreatic lipase has been immobilized on carboxyl-functionalized silica-coated magnetic nanoparticles. However, this method can reduce biocatalytic activity and viability due to the toxicity of coupling agents [130]. Additionally, weak bonds on carrier surfaces (e.g., chitosan nanofibers) increase the risk of the leakage of cells, making this method unsuitable for genetically modified microorganisms [131].

Adsorption, the simplest method, involves bacterial cells adhering to the surface of water-insoluble carriers via Van der Waals interactions, ionic forces, and hydrogen bonds [119,132,133]. This method is cost-effective, easy to operate, and preserves direct contact between microorganisms and contaminants. Biofilms, a natural form of bacterial immobilization through adsorption, further enhance the stability and functionality of immobilized systems [127].

## 5. Carriers Used for Bacterial Immobilization

Polymeric carriers (e.g., alginate, silica, chitosan, polyvinyl alcohol, etc.), chars (e.g., biochar and hydrochar), and nanocarriers (carbon nanotubes, cobalt nanoparticles, silica nanoparticles, etc.) play a significant role in microbial immobilization systems. They not only directly effect the viability of the microorganisms, but also effect the degradation efficacy processes [134,135,136,137,138,139].

For a successful biofilm formation, carriers should have certain characteristics (e.g., good porosity, high surface area, and various functional groups) that facilitate immobilization and do not cause toxicity on the bacterial cells [140]. Carriers should provide high biological and chemical stability and a large adhesion capacity for the cells. Moreover, materials that are supposed to convert into carriers should be inexpensive, easy to handle, and regenerative [33,141,142]. The main purpose of utilizing carriers is to provide a scaffold/structure for biological reactions without altering the native bacteria cell biological activity.

**Figure 3:**
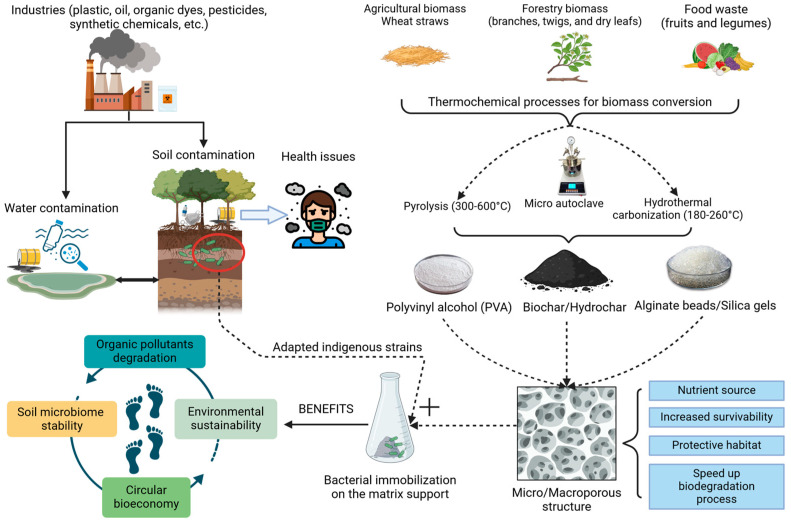
An overview of the main sources of organic pollutants and possible bioremediation strategies by using bacterial immobilization on a natural carrier.

To achieve suitable carriers for bacterial immobilization, biomass and thermochemical conversion processes should be considered [143]. Biomass from various sources (e.g., agricultural, forestry, and food) treated thermochemically (pyrolysis or hydrothermal carbonization) can provide a proper micro/macroporous structure for immobilization [119,144]. Thermochemical processes can break down the lignocellulosic molecules within the cellular wall of plants, facilitating pore formation and increasing the surface area. The new pores that are formed can facilitate immobilization efficacy and biofilm formation [145]. Some principles should be adhered to when using the obtained porous materials as carriers, as follows: a regenerative material, largely available, accessible, and inexpensive [146,147]. Such practices enable the maximum utilization of various substrates at each stage of waste generation and advance the implementation of circular bioeconomy (Figure 3).

The immobilization of bacterial cells on porous carriers/matrices can, in many cases, speed up the degradation rate, increase the bacterial survivability, and compensate with nutrients the metabolic mechanism of bacteria in nutrient-depleted environments [148]. These porous materials act as a “shield” in highly polluted environments [119,133]. Working with indigenous strains, identified in polluted environments, will reduce the alteration of the native microbiome [149]. Table 3 gives an overview for the degradation of organic pollutants using bacterial strains immobilized on various support materials/carriers.

As previously mentioned, carriers/support materials can be divided into two classes, according to their source and main composition—inorganic or organic [150]. In general, inorganic substrates have the advantage of being easy to find and having excellent mechanical strength compared with the organic carriers that have many functional groups and more stable structures. Organic carriers are also divided into natural carriers and synthetic polymers [119,120,131]. Both classes (inorganic and organic) of materials that are used in the immobilization of microorganisms offer a friendly solution for cleaning polluted sites.

A variety of inorganic materials (quartz, magnetite, volcanic rocks, vermiculite, porous glass, porous ceramic, silica-based materials, nanoparticles, carbon-based materials, etc.) have been successfully used in bacterial immobilization processes [151,152,153]. These types of carriers exhibit high chemical, physical, and biological resistance, although their small number of functional groups constitutes a disadvantage in bonding, and they usually need to be combined with natural polymers and synthetic nanoparticles [131,154,155,156,157].

Natural organic carriers (e.g., bagasse, vine shots, wheat straws, spruce bark, rice, diatomite alginate, κ-carrageenan, chitosan, alginate, charcoal, husks of sunflower seeds, plant fibers, corncob, mycelium, sawdust, etc.) have a large capacity for loading bacteria and provide high diffusion rates. They have a high number of hydrophilic bonds, pH stability, and biodegradability [148,158,159,160]. For assuring the bacterial colonization, carriers should have a specific macro-porous structure (>50 nm; IUPAC classification) [31,32]. This can be achieved by choosing the proper thermal conversion process; for example, hydrothermal carbonization (HTC), a cheap and simple method [38].

Synthetic polymers, including polyvinyl alcohol, polyvinyl chloride, polystyrene, and polyurethane foam, are mostly used in immobilization processes due to their versatile chemical properties, mechanical strength, and biocompatibility with microorganisms [131,161,162,163].

Novel support materials (nanocarriers) are emerging advanced support materials used in various areas, such as drug delivery, biocatalysis, and immobilization processes [164]. Due to their nanoscale dimensions, high surface area-to-volume ratio, and versatile properties for binding, they are ideal for many applications. Some types of nanocarriers include polymeric nanoparticles, silica nanoparticles, carbon nanotubes, cobalt nanoparticles, etc. [165,166,167].

**Table 3:** Degradation of organic pollutants using bacterial strains immobilized on various support materials/carriers. DDT = *dichlorodiphenyltrichloroethane*.

Carrier	Bacterial Genus	Experimental Conditions	Targeted Pollutant and Initial Concentration	Degradation Efficacy		References
				Non-Immobilized	Immobilized	
Biochar	*Bacillus*	Simulated sewage	Chlortetracycline (73.75 mg/L)	66%	83%	[168]
Polyvinyl alcohol–sodium alginate–kaolin	*Bacillus*	Synthetic medium	Trinitrotoluene (120 mg/L)	72%	99%	[169]
Modified peanut shell powder	*Mycobacterium*	Simulated polluted water	Pyrene (50 mg/L)	45%	70%	[170]
Rice straw biochar	*Mycobacterium*	Real contaminated soil	Phenanthrene (50 mg/L)	43%	69%	[171]
Nanocellulose fibers	*Arthrobacter*	Spiked medium	Diuron (10 mg/L)	86%	99%	[167]
Expanded polystyrene	*Arthrobacter*	Synthetic medium	Pentane (50 mL/500 mL)	70%	90%	[172]
Sunflower seeds husk	*Rhodococcus*	Real soil	Crude oil (25 g/kg)	28%	66%	[173]
Magnetic nanoparticles	*Rhodococcus*	Spiked medium	Chlorophenol (0.50 mM)	50%	80%	[166]
Sodium alginate + polyvinyl alcohol	*Flavobacterium*	Real soil	Ammonia nitrogen (50 mg/L)	25%	>80%	[174]
Polyurethane	*Flavobacterium*	Spiked medium	Pentachloro-phenol 300 (mg/L)	20%	90%	[175]
Polyvinyl alcohol	*Acinetobacter*	Spiked medium	Phenol (1100 mg/L)	50%	>99%	[176]
Membrane Bioreactor	*Pseudomonas*	Real wastewater	Phenanthrene (20 mg/L)	77%	96%	[177]
Alginate, agar, and polyacrylamide	*Pseudomonas*	Spiked medium	Ethylbenzene (1 mL/L)	2%	60%	[178]
Modified biochar	*Pseudomonas*	Real wastewater	Triclocarban (10 mg/L)	32%	80%	[179]
Bamboo charcoal and wood charcoal	Bacterial consortium	Real wastewater	Nonylphenol (50 mg/L)	20%	70%	[180]
Coco-peat and rice hull powder	Bacterial consortium	In vitro sea water	DDT (pesticide) (0.75 mL/150 mL)	25%	86%	[181]

## 6. Conclusions, Challenges, and Perspectives

Immobilized bacteria on various support matrices offer significant advantages in the bioremediation of sites contaminated with toxic organic compounds. The main benefits of immobilization are enhanced stability/viability and degradation efficacy, compared to planktonic cells. The support matrix acts as a sink for pollutants and as a “shelter” for the bacterial cells in highly polluted environments, prolonging their metabolic activity and improving degradation rates. Additionally, immobilized bacterial systems allow for the controlled release of bacteria at contaminated sites, ensuring a safe and targeted application that minimizes the risk of bacterial dispersion and potential ecological impacts. By localizing bacterial activity, immobilization also prevents the spread of contaminants, which is particularly beneficial for sites with high concentrations of toxic compounds where containment is crucial. The importance of high porosity, surface area, and functional groups in carriers, as well as their nontoxicity and stability, are emphasized. In recent years, synthetic polymers and nanocarriers have emerged as versatile alternatives.

The thermal conversion of biomass materials can produce effective carriers for bacterial immobilization, but challenges such as toxic by-products (e.g., PAHs and VOCs) and pollutant release during biochar/hydrochar aging remain significant concerns. These issues can impact bacterial viability and long-term bioremediation efficiency. Optimizing thermal conversion parameters such as porosity, surface area, and functionality while monitoring chars’ stability, we can cope with some of the long-term challenges of this biotechnology.

In comparison, nanocarriers stand out for their nanoscale dimensions and high efficiency, with examples such as Fe_3_O_4_-coated nanoparticles achieving 30% greater efficiency over free cells. Combining immobilized bacteria with other remediation technologies, such as phytoremediation or chemical oxidation, could offer synergistic effects and improve overall efficiency, particularly for complex contamination sites.

Therefore, this paper summarizes the sources of organics pollutants, as well as their effects on the environment and human health.

## Figures and Tables

**Table 2:** Main immobilization methods for microbial cells with their costs, benefits, and side effects.

Type of Immobilization	Costs	Benefits	Potential Problems	References
Entrapment	Low costs due to the manufacturing process and materials	Prevents leaking of cells into the environment; diffusion of pollutants and various metabolic products is facilitated by the porous structure of the matrix; protective barrier against pollutants	Leaking effects when the pores are larger than the immobilized cells; requires high costs of maintenance; limits the exchange of nutrients with the exterior environment	[120,122]
Encapsulation	Moderate costs, depends on the materials and processes used	Prevents biocatalyst leakage; has a long-term effectiveness; can be tailored for specific pollutants	High risks of leaching in case of improper encapsulation of polymeric gels or other materials	[123,124]
Covalent binding	High costs due to the materials and the applied processes	Durable solution; strong covalent bonds that prevent the leaking of molecules into the environment	Can often be toxic and effect cell viability or enzyme activity	[125,126]
Adsorption	Low costs due to cost of materials and processes applied	Absorbents can be regenerated and reused; does not require chemical additives	High risk of leakage from the matrix due to weak binding forces and unstable interactions	[127,128]

## Data Availability

The original contributions presented in the study are partially included in the article, and further inquiries can be directed to the corresponding author.
